# Immunogenicity of a bivalent BA.1 COVID-19 booster vaccine in people with HIV in the Netherlands

**DOI:** 10.1097/QAD.0000000000003933

**Published:** 2024-05-28

**Authors:** Marlou J. Jongkees, Ngoc H. Tan, Daryl Geers, Rory D. de Vries, Corine H. GeurtsvanKessel, Kathryn S. Hensley, Roos S.G. Sablerolles, Susanne Bogers, Lennert Gommers, Blerdi Blakaj, Pedro Miranda Afonso, Bettina E. Hansen, Bart J.A. Rijnders, Kees Brinkman, P. Hugo M. van der Kuy, Anna H.E. Roukens, Casper Rokx

**Affiliations:** a Department of Internal Medicine, Section Infectious Diseases, and Department of Medical Microbiology and Infectious Diseases; b Department of Hospital Pharmacy; c Department of Viroscience; d Department of Medical Oncology; e Department of Biostatistics and Department of Epidemiology, Erasmus University Medical Centre, Rotterdam, the Netherlands; f Institute of Health Policy, Management and Evaluation, University of Toronto; g Toronto Centre for Liver Disease, Toronto General Hospital University Health Network, Toronto, Canada; h Department of Internal Medicine and Infectious Diseases, OLVG Hospital, Amsterdam; i Department of Infectious Diseases, Leiden University Medical Centre, Leiden, the Netherlands.

**Keywords:** cellular immunity, combined vaccines, COVID-19 serological testing, COVID-19 vaccines, cytokines, HIV, SARS-CoV-2

## Abstract

**Objective:**

We evaluated the immunogenicity of a bivalent BA.1 COVID-19 booster vaccine in people with HIV (PWH).

**Design:**

Prospective observational cohort study.

**Methods:**

PWH aged ≥45 years received Wuhan-BA.1 mRNA-1273.214 and those <45 years Wuhan-BA.1 BNT162b2. Participants were propensity score-matched 1 : 2 to people without HIV (non-PWH) by age, primary vaccine platform (mRNA-based or vector-based), number of prior COVID-19 boosters and SARS-CoV-2 infections, and spike (S1)-specific antibodies on the day of booster administration. The primary endpoint was the geometric mean ratio (GMR) of ancestral S1-specific antibodies from day 0 to 28 in PWH compared to non-PWH. Secondary endpoints included humoral responses, T-cell responses and cytokine responses up to 180 days post-vaccination.

**Results:**

Forty PWH received mRNA-1273.214 (*N* = 35) or BNT162b2 (*N* = 5) following mRNA-based (*N* = 29) or vector-based (*N* = 11) primary vaccination. PWH were predominantly male (87% vs. 26% of non-PWH) and median 57 years [interquartile range (IQR) 53–59]. Their median CD4^+^ T-cell count was 775 (IQR 511–965) and the plasma HIV-RNA load was <50 copies/ml in 39/40. The GMR of S1-specific antibodies by 28 days post-vaccination was comparable between PWH [4.48, 95% confidence interval (CI) 3.24–6.19] and non-PWH (4.07, 95% CI 3.42–4.83). S1-specific antibody responses were comparable between PWH and non-PWH up to 180 days, and T-cell responses up to 90 days post-vaccination. Interferon-γ, interleukin (IL)-2, and IL-4 cytokine concentrations increased 28 days post-vaccination in PWH.

**Conclusion:**

A bivalent BA.1 booster vaccine was immunogenic in well treated PWH, eliciting comparable humoral responses to non-PWH. However, T-cell responses waned faster after 90 days in PWH compared to non-PWH.

## Introduction

People with HIV (PWH) showed a comparatively lower antibody response following primary coronavirus disease 2019 (COVID-19) vaccination than people without HIV (non-PWH) [1–4]. This is particularly true for PWH with a CD4^+^ T-cell count < 250 cells/μl, men and older individuals [4]. Although various analyses reported different impacts of COVID-19 vaccination on PWH compared to non-PWH, larger-scale studies showed that reduced vaccine immunogenicity correlated with more breakthrough infections [5] and severe COVID-19 in vaccinated PWH [6,7].

Waning immunity, which reduces vaccine effectiveness [8], led to the use of COVID-19 booster vaccines in the general public. Regarding the immune escape viral variant omicron (B.1.1.529), booster vaccines based on the ancestral strain continued to protect against severe COVID-19, but were largely ineffective in preventing infection [9–11]. This led the World Health Organization (WHO) to recommend the introduction of omicron-containing mRNA-based vaccines as boosters, a policy which was adopted in the Netherlands in Q4 2022 [12].

In PWH, a first (monovalent) booster improved neutralizing antibody responses, led to seroconversion in primary non-responders, and reduced the risk of severe acute respiratory syndrome coronavirus 2 (SARS-CoV-2)-related infections and mortality [13,14]. However, seroconversion rates remained slightly lower than in non-PWH [15]. Furthermore, PWH were excluded from the registration studies showing that BA.1-adapted bivalent vaccines induced superior neutralizing responses compared to monovalent booster vaccines [16,17].

Thus far, immunogenicity of a bivalent BA.1 booster vaccine in PWH compared to non-PWH has not been studied. However, this information is pivotal for designing booster vaccination policies for PWH in terms of possible adjustments to the COVID-19 vaccination schedule recommended for the general public. We hypothesized that the SARS-CoV-2-specific bivalent BA.1 vaccine response would be comparable between well treated PWH and non-PWH. The COVID-19 additional booster vaccination in PWH study (COVIH-BOOST-2) aimed to compare the immunogenicity of a bivalent BA.1 booster vaccine in PWH and non-PWH.

## Methods

### Study design and participants

This observational cohort study was conducted in two academic hospitals in the Netherlands. Participants were recruited between October and November 2022 and followed-up for 180 days. The inclusion criteria were a minimum age of 18 years, a confirmed HIV infection and no COVID-19 vaccination or documented SARS-CoV-2 infection in the preceding 3 months. There were no requirements regarding the number of prior COVID-19 vaccinations or SARS-CoV-2 infections. Non-PWH were recruited from the SWITCH-ON trial, which assessed the immunogenicity of a bivalent BA.1 booster vaccine in Dutch healthcare workers [18]. The COVIH-BOOST-2 study design and inclusion criteria were similar to those of the SWITCH-ON trial. PWH were propensity score-matched 1 : 2 to the nearest non-PWH neighbour by age, primary vaccination regimen (mRNA-based or vector-based), history of SARS-CoV-2 infection, number of prior COVID-19 boosters, and the level of spike (S1)-specific antibodies on day 0 before the bivalent BA.1 booster vaccine was administered.

This study adhered to the Strengthening the Reporting of Observational Studies in Epidemiology (STROBE) guideline to ensure comprehensive reporting of the data (Table 1, Supplemental Digital Content).

### Clinical procedures

Participants aged 45 years and above received 50 μg of the bivalent Wuhan-BA1 mRNA-1273.214 vaccine while those under 45 years received 30 μg of the bivalent Wuhan-BA.1 BNT162b2 vaccine, as per national guidelines [19]. Blood samples were obtained on days 0, 7, 28, 90 and 180 for collection of serum and peripheral blood mononuclear cells (PBMCs). Clinical data were collected in an electronic case record file and included year of birth, sex assigned at birth, ethnicity, current use of combination antiretroviral therapy (cART), most recent plasma HIV-RNA load (copies/ml), most recent CD4^+^ T-cell count (cells/μl), nadir CD4^+^ T-cell count (cells/μl), comorbidities, co-medication, prior COVID-19 vaccinations and history of SARS-CoV-2 infection. Testing behaviour and breakthrough infections were evaluated with questionnaires and measurement of nucleocapsid (N)-specific SARS-CoV-2 antibodies. Solicited reactions and medication use in the first seven days after the bivalent BA.1 vaccine administration were evaluated using printed diaries.

### Laboratory procedures

#### Humoral responses

The concentrations of IgG binding antibodies specific for the ancestral spike protein S1 subunit were measured using a validated quantitative chemiluminescence immunoassay, the LIAISON SARS-CoV-2 TrimericS IgG assay (DiaSorin, Saluggia, Italy), with a lower limit of quantification of 4.81 BAU/ml, at the Erasmus University Medical Centre. SARS-CoV-2 N-specific antibodies were measured on days 0, 90 and 180 using the Abbott SARS-CoV-2 immunoglobulin G (IgG) assay, with a ≥1.4 signal-to-cut-off ratio for positivity, to identify unreported SARS-CoV-2 infections.

#### Peripheral blood mononuclear cell isolations

PBMCs were isolated by density gradient centrifugation (Ficoll-Hypaque, GE Healthcare Life Sciences) and collected in RPMI-1640 (Life Technologies) supplemented with 3% foetal bovine serum (FBS). PBMCs were washed three times before counting. Cells were frozen in freezing media (90% FBS supplemented with 10% dimethyl sulfoxide) and stored in liquid nitrogen until use.

#### T-cell responses

SARS-CoV-2-specific T-cell responses were assessed by measuring interferon (IFN)-γ concentrations after stimulating whole blood using the commercially available IFN-γ release assay (IGRA; QuantiFERON SARS-CoV-2 kit containing three antigenic stimulation pools (certified for IVD use), QIAGEN, Hilden, Germany). Heparinized whole blood was incubated with the SARS-CoV-2 antigens (antigens 1, 2 and 3) for 20–24 h at 37°C. As positive and negative controls, mitogen- and carrier (NIL)-coated tubes were used, respectively. After incubation, plasma was obtained by centrifugation, and IFN-γ production in response to antigen stimulation was measured by ELISA (QIAGEN). Results were expressed in international units (IU) IFN-γ/ml after subtracting the NIL control values as interpolated from a standard calibration curve. The lower limit of detection was 0.01 IU/ml, and the cut-off level for positivity was 0.15 IU/ml.

#### Cytokine responses

The SARS-CoV-2-specific cytokine response was measured using a human T helper cytokine panel (LEGENDplex, Biolegend, CA, USA), which profiled 12 different cytokines [interleukin (IL)-2, IL-4, IL-5, IL-6, IL-9, IL-10, IL-13, IL-17A, IL-17F, IL-22, IFN-γ, and tumour necrosis factor (TNF)-α] in plasma that was obtained from whole blood stimulated with peptides of the SARS-CoV-2 spike-protein using the IGRA as mentioned and stored at −80°C until use. After thawing on ice, plasma samples were centrifuged and two-fold dilutions were prepared and incubated for 2 h with monoclonal capture antibody-coated beads. Following the first incubation, the beads were washed twice and incubated for 1 h with biotin-labelled detection antibodies and afterwards with streptavidin-PE for 30 min. Then, samples were washed twice and stained. After staining, beads were acquired by flow cytometry on a BD FACSLyric with FlowJo software (BD Bioscience, NJ, USA). The data obtained were analysed with LEGENDplex V8.0 software (BioLegend). The quantity of each cytokine was calculated based on the intensity of the streptavidin-PE signal and a freshly prepared standard curve. Results were expressed in picogram (pg) cytokine/ml after subtraction of the NIL control value.

### Outcomes

The primary outcome was the geometric mean ratio (GMR) of S1-specific antibodies between day 0 and 28 after a bivalent BA.1 booster vaccine in PWH compared to non-PWH. The secondary outcomes included the humoral and T-cell responses in PWH and non-PWH on days 0, 7, 28, 90 and 180, and the cytokine responses in PWH on days 0, 28 and 180. The exploratory outcomes were humoral and T-cell responses stratified by type of primary vaccination regimen (mRNA-based vs. vector-based), reported history of SARS-CoV-2 infection (yes vs. no), and by the found cytokine cluster types, as well as the association between S1-specific antibodies and CD4^+^ T-cell count in PWH. Lastly, vaccine-solicited reactions, scored as mild (symptoms present, no functional impairment or need for medication), moderate (need for medication, no functional impairment), or severe (impaired daily functioning), were evaluated.

### Sample size and statistical analysis plan

To detect a difference of 0.25 in the GMR of S1-specific antibodies, assuming a 0.372 standard deviation, including 29 PWH matched 1 : 2 to 58 non-PWH resulted in 80% power at a two-sided alpha of 5%. The sample size calculation was performed using the R package pwr (v.1.3-0). A 1 : 2 matching ratio was chosen to increase precision of the estimated effect without a commensurate increase in bias [20]. Propensity score-matching between PWH and non-PWH was performed using the R package MatchIt (v.4.5.5).

Data were described using count (percentage) or median [interquartile range (IQR)]. Geometric mean titres (GMTs) or geometric means (GMs) and GMRs of S1-specific antibodies and IFN-γ levels were reported with 95% confidence intervals (CIs) and compared between PWH and non-PWH using independent *t*-tests. GMTs and GMs were calculated as the mean of logarithmically transformed results and GMRs as the mean of the difference in logarithmically transformed results, and all means were exponentiated back to express results on the original scale. Humoral and T-cell responses were compared between mRNA-based and vector-based primary vaccinations, and between participants with hybrid immunity (vaccination and a documented SARS-CoV-2 infection) and those with vaccine-induced immunity alone by independent *t*-tests. To investigate the association of CD4^+^ T-cell count with S1-specific antibodies, a Spearman rank correlation was performed. Comparisons of cytokine concentrations from day 0–28, 0–180 and 28–180 were performed by Wilcoxon matched-pairs signed rank tests. An unsupervised clustering of the centred and scaled SARS-CoV-2-specific cytokine concentrations IFN-γ, IL-2, IL-4, IL-5 and IL-13 was performed, using the Ward's method, and a heatmap of the cluster analysis was created using the R package pheatmap (1.0.12). Humoral and T-cell responses were compared between the found clusters by independent *t*-tests. *P*-values <0.05 were considered statistically significant. Undetectable S1-specific antibodies (<4.81 BAU/ml) were reported as 4.81 in the statistical analyses, IFN-γ levels (<0.01 IU/ml) as 0.01 and cytokine concentrations (<0.001 pg/ml) as 0.001. Participants with a SARS-CoV-2 infection were censored from further analysis at time points after the date of the infection. Data were analysed using GraphPad Prism version 10.2.1 and RStudio version 4.2.1.

### Ethics committee approval

The trial was performed in accordance with the Declaration of Helsinki, Good Clinical Practice guidelines and the Dutch Medical Research Involving Human Subjects Act (WMO). The trial was approved by the Medical Ethics Committees United Nieuwegein (MEC-U, reference 20.125) and registered on the International Clinical Trials Platform (EUCTR2021-001054-57-N). All participants signed an informed consent form.

## Results

### Baseline characteristics

Between October 13 and November 29, 2022, 41 PWH were enrolled. One participant was excluded due to a SARS-CoV-2 breakthrough infection before the primary endpoint evaluation. Of the 40 included PWH, five participants were younger than 45 years, receiving BNT162b2, and 35 participants were at least 45 years, receiving mRNA-1273.214. The baseline characteristics of PWH and non-PWH are described in Table 1. PWH had a median age of 57 years (IQR 52–63), with a most recent and nadir median CD4^+^ T-cell count of 775 (IQR 511–965) and 270 (IQR 180–348), respectively. All participants received cART and all but one had a suppressed plasma HIV-RNA load of < 50 copies/ml. Eleven (27%) PWH had received two doses of ChAdOx1-S, 25 (63%) two doses of BNT162b2, and 4 (10%) two doses of mRNA-1273 as primary vaccination regimen. After matching, the frequency of mRNA-based or vector-based primary vaccine platform use was similar in PWH and non-PWH. Of those with an mRNA-based primary regime, BNT162b2 was more often used in PWH. Of those with a vector-based primary regime, PWH exclusively received ChAdOx1.S, while all non-PWH received Ad26.COV2.S. The number of COVID-19 booster vaccines and S1-specific antibodies at baseline (day 0) were comparable among both groups. PWH were more often male (87% vs. 26% of non-PWH), slightly older (57 vs. 54 years in non-PWH), had a lower incidence of prior SARS-CoV-2 infection, and had less frequently detectable N-specific antibodies (10% vs. 16% in non-PWH). Seven PWH seroconverted to N-specific antibodies during the 180 days follow-up (of whom one reported a positive COVID-19 antigen test), and 19 non-PWH seroconverted (of whom 13 reported a positive COVID-19 antigen test).

**Table 1:** Baseline characteristics of participants stratified based on the primary vaccination regimen.

	People with HIV			People without HIV		
	Overall	mRNA-based prime	Vector-based prime	Overall	mRNA-based prime	Vector-based prime
	*N* = 40	*N* = 29 (73%)	*N* = 11 (27%)	*N* = 80	*N* = 58 (73%)	*N* = 22 (27%)
Sex assigned at birth						
Male	35 (87%)	24 (83%)	11 (100%)	21 (26%)	11 (19%)	10 (45%)
Female	5 (13%)	5 (17%)	0	59 (74%)	47 (81%)	12 (55%)
Age, years	57 (52–63)	55 (49–59)	63 (62–65)	54 (50–57)	53 (42–56)	55 (54–57)
On cART						
Yes	40 (100%)	29 (100%)	11 (100%)	NA	NA	NA
Most recent plasma HIV viral load						
<50 copies/ml	39 (98%)	28 (97%)	11 (100%)	NA	NA	NA
≥50 copies/ml	1 (2%)	1 (3%)	0	NA	NA	NA
Most recent CD4^+^ T-cell count						
<250 cells/μl	1 (2%)	1 (3%)	0	NA	NA	NA
250–500 cells/μl	8 (20%)	7 (24%)	1 (9%)	NA	NA	NA
>500 cells/μl	31 (78%)	21 (73%)	10 (91%)	NA	NA	NA
Nadir CD4^+^ T-cell count						
<250 cells/μl	17 (44%)	12 (42%)	5 (46%)	NA	NA	NA
250–500 cells/μl	13 (32%)	11 (38%)	2 (18%)	NA	NA	NA
>500 cells/μl	6 (15%)	3 (10%)	3 (27%)	NA	NA	NA
Unknown	4 (9%)	3 (10%)	1 (9%)	NA	NA	NA
Ethnicity						
African	2 (5%)	2 (7%)	0	0	0	0
Asian	2 (5%)	2 (7%)	0	3 (4%)	3 (5%)	0
European	34 (85%)	24 (83%)	10 (91%)	74 (92%)	52 (90%)	22 (100%)
North American	0	0	0	1 (1%)	1 (2%)	0
South American	2 (5%)	1 (3%)	1 (9%)	0	0	0
Other	0	0	0	2 (3%)	2 (3%)	0
Comorbidities^a^						
Cardiovascular diseases	3 (7%)	1 (3%)	2 (18%)	1 (1%)	1 (2%)	0
Pulmonary diseases	3 (7%)	2 (7%)	1 (9%)	4 (5%)	3 (5%)	1 (5%)
Diabetes	1 (2%)	1 (3%)	0	1 (1%)	1 (2%)	0
Liver diseases	8 (20%)	5 (17%)	3 (27%)	1 (1%)	1 (2%)	0
Kidney diseases	3 (7%)	3 (10%)	0	1 (1%)	1 (2%)	0
None	26 (65%)	21 (72%)	5 (45%)	72 (91%)	51 (87%)	21 (95%)
Primary vaccination regimen						
BNT162b2	25 (62%)	25 (86%)	0	29 (36%)	29 (50%)	0
mRNA-1273	4 (10%)	4 (14%)	0	29 (36%)	29 (50%)	0
ChAdOx1-S	11 (28%)	0	11 (100%)	0	0	0
Ad26.COV2.S	0	0	0	22 (28%)	0	22 (100%)
Number of booster doses^b^						
0	2 (5%)	1 (3%)	1 (9%)	0	0	0
1	20 (50%)	19 (66%)	1 (9%)	59 (74%)	55 (95%)	4 (18%)
2	17 (43%)	8 (28%)	9 (82%)	21 (26%)	3 (5%)	18 (82%)
3	1 (2%)	1 (3%)	0	0	0	0
History of SARS-CoV-2 infection						
No	21 (52%)	15 (52%)	6 (55%)	23 (29%)	16 (28%)	7 (32%)
Yes, once	19 (48%)	14 (48%)	5 (45%)	56 (70%)	41 (70%)	15 (68%)
Yes, twice	0	0	0	1 (1%)	1 (2%)	0
Combined number of booster doses and SARS-CoV-2 infections						
1	14 (35%)	12 (41%)	2 (18%)	15 (19%)	14 (24%)	1 (5%)
2	17 (43%)	12 (41%)	5 (45%)	51 (64%)	42 (73%)	9 (40%)
3	8 (20%)	4 (15%)	4 (37%)	14 (17%)	2 (3%)	12 (55%)
4	1 (2%)	1 (3%)	0	0	0	0
Geometric mean of S1-specific antibodies on day 0, BAU/ml	3431 (2459–4787)	4180 (2775–6298)	2039 (1229–3382)	3349 (2620–4282)	4222 (3177–5612)	1819 (1203–2750)
Nucleocapsid on day 0						
Negative	36 (90%)	26 (90%)	10 (91%)	67 (84%)	46 (79%)	21 (95%)
Positive	4 (10%)	3 (10%)	1 (9%)	13 (16%)	12 (21%)	1 (5%)
Time between last booster and bivalent BA.1 booster vaccination, days	275 (168–298)	287 (178–299)	195 (151–236)	303 (266–310)	305 (298–310)	266 (263–268)

Values are count (%), median (IQR), or geometric mean (95% CI).

a Percentages may not sum to 100 due to variations in the number of comorbidities per participant.

b Booster doses are the number of vaccines administered after a completed primary vaccination regimen with two doses of BNT162b2, mRNA-1273, or ChAdOx1-S or one dose of Ad26.COV2.S.

Abbreviations: BAU, binding antibody units; cART, combination antiretroviral therapy; CI, confidence interval; HIV, human immunodeficiency virus; IQR, interquartile range; NA, not applicable.

### Humoral responses

**Fig. 1:**
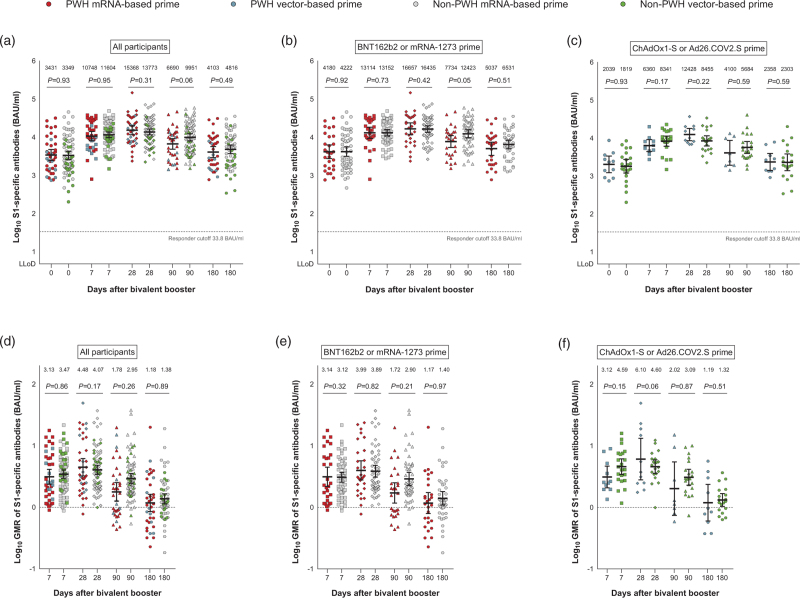
SARS-CoV-2 spike (S1)-specific antibody response after bivalent BA.1 booster vaccination.

S1-specific antibody responses up to 180 days after bivalent BA.1 booster vaccination were comparable between PWH and non-PWH (Fig. 1a). The GMTs of S1-specific antibodies increased from 3431 (95% CI 2459–4787) in PWH and 3349 (95% CI 2620–4282) in non-PWH on day 0 to 15 368 (95% CI 11 684–20 213) in PWH and 13 773 (95% CI 11 544–16 434) in non-PWH on day 28 after bivalent BA.1 booster vaccination. The GMRs of S1-specific antibodies in PWH vs. non-PWH compared to day 0 were 4.48 (95% CI 3.24–6.19) vs. 4.07 (95% CI 3.42–4.83) on day 28, and 1.18 (95% CI 0.85–1.63) vs. 1.38 (95% CI 1.14–1.66) on day 180 (Fig. 1d). Within the subgroups with an mRNA-based or vector-based primary vaccination, GMTs and GMRs of S1-specific antibodies were not statistically different between PWH and non-PWH (Fig. 1b, c, e, f).

(a) Detection of ancestral S1-specific antibodies at baseline before bivalent BA.1 booster vaccination (day 0, indicated by circles) and on days 7 (squares), 28 (diamonds), 90 (triangles) and 180 (hexagons) after bivalent BA.1 booster vaccination in propensity score-matched PWH (*N* = 40) and non-PWH (*N* = 80) and in participants with an mRNA-based prime (*N* = 29 PWH and *N* = 58 non-PWH; panel b) or vector-based prime (*N* = 11 PWH and *N* = 22 non-PWH; panel c). The numbers above the plots and the middle whiskers in the plot indicate the geometric mean titres per time point, while the lower and upper whiskers in the plot indicate the corresponding 95% confidence intervals. The lower limit of detection was set at 4.81 BAU/ml, and the cut-off responder value was set at 33.8 BAU/ml (horizontal dashed line). Comparisons between groups were performed using the independent *t*-test. (d) GMRs of S1-specific antibodies compared to day 0 on days 7, 28, 90 and 180 in all participants, and in participants with an mRNA-based prime (panel e) or vector-based prime (panel f). The horizontal dashed line indicates a Log_10_ GMR of 0, corresponding to no difference in S1-specific antibodies. Comparisons between groups were performed using the independent *t*-test. Data from four participants on day 90 and two participants on day 180 are missing. Abbreviations: BAU, binding antibody units; GMR, geometric mean ratio; LLoD, lower limit of detection; non-PWH, people without human immunodeficiency virus; PWH, people with human immunodeficiency virus; S, spike.

Within PWH, the GMRs of S1-specific antibodies compared to day 0 were comparable until day 180 between those who received an mRNA-based or vector-based primary vaccination. However, the S1-specific antibodies were numerically lower in PWH with vector-based compared to mRNA-based primary vaccination at all five study visits, *P* = 0.007 on day 7 and *P* = 0.06 on day 180 (Figure 1, Supplemental Digital Content). No association was found between the most recent CD4^+^ T-cell count and the S1-specific antibodies on day 28 (Spearman *r* = 0.12, *P* = 0.46).

### T-cell responses

**Fig. 2:**
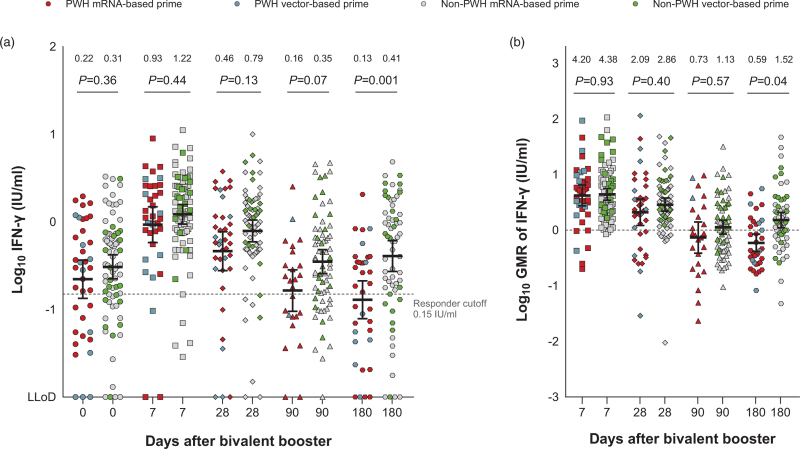
SARS-CoV-2-specific T-cell response after bivalent BA.1 booster vaccination.

The GM of the IFN-γ levels after the stimulation of whole blood was comparable on day 28 between PWH (0.46, 95% CI 0.63–1.27) and non-PWH (0.79, 95% CI 1.01–1.70); see Fig. 2a. The GM of the IFN-γ levels and GMRs of the IFN-γ levels compared to day 0 were comparable between PWH and non-PWH up to day 90 (Fig. 2b). On day 180, the GM of IFN-γ was lower in PWH (0.13, 95% CI 0.08–0.21) than in non-PWH (0.41, 95% CI 0.27–0.61). This observation of a lower GM on day 180 was similar across the matched groups with mRNA-based or vector-based primary vaccination (Figure 2, Supplemental Digital Content).

(a) Detection of IFN-γ (IU/ml) after the stimulation of whole blood with antigen 2 in coated QuantiFERON tubes at baseline before bivalent BA.1 booster vaccination (0 days, circles) and on days 7 (squares), 28 (diamonds), 90 (triangles) and 180 (hexagons) after bivalent BA.1 booster vaccination in propensity score-matched PWH (*N* = 40) and non-PWH (*N* = 80). The numbers above the plots and the middle whiskers in the plot indicate the geometric means per time point, while the lower and upper whiskers in the plot indicate the corresponding 95% confidence intervals. The lower limit of detection was set at 0.01 IU/ml, as per the manufacturer's instructions, and the cut-off responder value was set at 0.15 IU/ml (horizontal dashed line). Comparisons between groups were performed using the independent *t*-test. (b) GMRs of IFN-γ compared to day 0 on days 7, 28, 90 and 180. The horizontal dashed line indicates a Log_10_ GMR of 0, corresponding to no difference in IFN-γ. Comparisons between groups were performed using the independent *t*-test. Data from two participants on all time points, 14 participants on day 90 and six participants on day 180 are missing. Abbreviations: GMR, geometric mean ratio; IU, international units; LLoD, lower limit of detection; non-PWH, people without human immunodeficiency virus; PWH, people with human immunodeficiency virus.

**Fig. 3:**
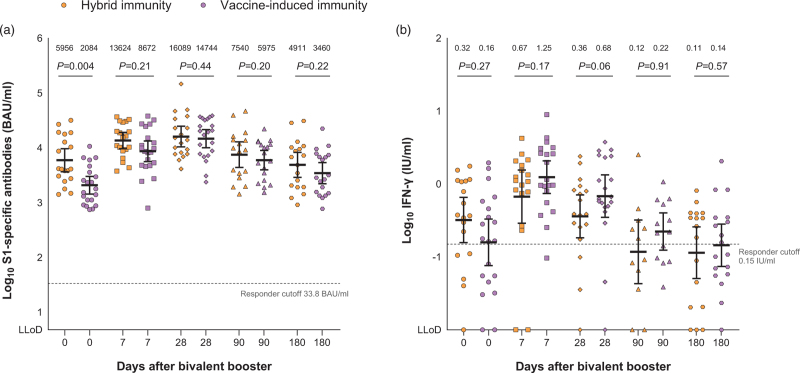
SARS-CoV-2 spike (S1)-specific antibody response and SARS-CoV-2-specific T-cell response in PWH with hybrid immunity versus vaccine-induced immunity alone.

Within the group of PWH, the GMs and GMRs of IFN-γ were comparable between PWH with an mRNA-based or vector-based primary vaccination. IFN-γ levels after stimulation with antigen 2 correlated well with the levels after stimulation with antigen 1 (*r* = 0.73, *P* < 0.0001) or antigen 3 (*r* = 0.83, *P* < 0.0001); see Figure 3, Supplemental Digital Content.

### Hybrid immunity

PWH with hybrid immunity showed higher levels of S1-specific antibodies on day 0 than PWH with vaccine-induced immunity alone (*P* = 0.004). This difference resolved after bivalent BA.1 booster vaccination (Fig. 3a). No significant differences in IFN-γ levels were found between PWH with hybrid immunity or vaccine-induced immunity alone (Fig. 3b).

(a) Detection of ancestral S1-specific antibodies at baseline before bivalent BA.1 booster vaccination (day 0, indicated by circles) and on days 7 (squares), 28 (diamonds), 90 (triangles) and 180 (hexagons) after bivalent BA.1 booster vaccination in PWH with vs. without a documented history of SARS-CoV-2 infection (*N* = 19 with hybrid immunity and *N* = 21 with vaccine-induced immunity alone). The lower limit of detection was set at 4.81 BAU/ml, and the cut-off responder value was set at 33.8 BAU/ml (horizontal dashed line). The numbers above the plots and the middle whiskers in the plot indicate the geometric mean titres per time point, while the lower and upper whiskers in the plot indicate the corresponding 95% confidence intervals. Comparisons of time points were performed using the independent *t*-test. (b) Detection of IFN-γ (IU/ml) after the stimulation of whole blood with overlapping spike protein peptide pools (antigen 2) in coated QuantiFERON tubes at baseline before bivalent BA.1 booster vaccination (day 0, indicated by circles) and on days 7 (squares), 28 (diamonds), 90 (triangles) and 180 (hexagons) after bivalent BA.1 booster vaccination in PWH with vs. without a documented history of SARS-CoV-2 infection (*N* = 19 with hybrid immunity and *N* = 21 with vaccine-induced immunity alone). The lower limit of detection was set at 0.01 IU/ml, as per the manufacturer's instructions, and the cut-off responder value was set at 0.15 IU/ml (horizontal dashed line). The numbers above the plots and the middle whiskers in the plot indicate the geometric means per time point, while the lower and upper whiskers in the plot indicate the corresponding 95% confidence intervals. Comparisons of time points were performed using the independent *t*-test. Abbreviations: BAU, binding antibody units; IU, international units; LLoD, lower limit of detection; non-PWH, people without human immunodeficiency virus; PWH, people with human immunodeficiency virus; S, spike.

### Cytokine responses

**Fig. 4:**
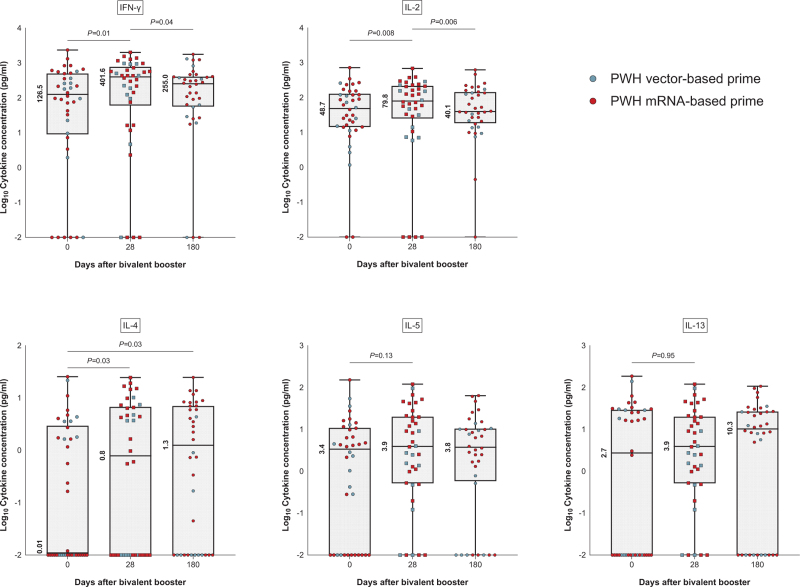
SARS-CoV-2-specific cytokine response in PWH after bivalent BA.1 vaccination.

An increase in the SARS-CoV-2-specific cytokine concentrations of IFN-γ, IL-2 and IL-4 was observed 28 days after bivalent BA.1 booster vaccination in PWH, while the cytokine concentrations of IL-5 and IL-13 did not differ between day 0 and day 28 (Fig. 4). An overview of the seven additional measured cytokines is shown in Figure 4, Supplemental Digital Content, revealing no major differences over time. The clustering analysis of the cytokine concentrations distinguished three different cluster types in PWH (Figure 5, Supplemental Digital Content). Cluster 1 was characterized by the highest concentrations of Th_1_-type and Th_2_-type cytokines, while cluster 2 and cluster 3 were characterized by lower cytokine concentrations of both the Th_1_-type and Th_2_-type. SARS-CoV-2-specific production of plasma cytokines was very limitedly detected in cluster 3 participants. Apart from a lower proportion of mRNA-primed individuals in cluster 3 (61%) compared to cluster 1 (88%), participants’ characteristics were comparable between the clusters. S1-specific antibody responses and T-cell responses on day 28 were not different among participants across the three clusters (Figure 6, Supplemental Digital Content).

Cytokine concentrations (pg/ml) for five different cytokines measured after stimulation of whole blood with antigen 2 in coated QuantiFERON tubes at baseline before bivalent BA.1 booster vaccination and on day 28 and 180 after bivalent BA.1 vaccination in all PWH (*N* = 40). The first row depicts the levels of the Th_1_-type cytokines IFN-γ and IL-2 and the second row depicts the levels of the Th_2_-type cytokines IL-4, IL-5 and IL-13. The horizontal lines within the whiskers and numbers indicate the medians, while the tops and bottoms indicate the interquartile ranges. Wilcoxon matched-pairs signed rank tests were applied to the comparisons. Data from two participants on all three time points and two participants on day 180 are missing. Abbreviations: IFN, interferon; IL, interleukin; pg, picogram; PWH, people with human immunodeficiency virus; Th, T helper.

### Solicited reactions

The bivalent BA.1 booster vaccine was well tolerated, and no serious adverse events were reported. Solicited reactions were generally mild in severity, and the most frequently reported reaction was pain at the injection site (58%; Table 2, Supplemental Digital Content).

## Discussion

Here, we show that bivalent BA.1 COVID-19 booster vaccination in PWH on cART and with a generally normal CD4^+^ T-cell count had an immunogenicity comparable to control participants without HIV. As expected, no vaccine-induced serious adverse events were found in this small group of participants.

To our knowledge, only two other studies reported on the immunogenicity of a bivalent booster vaccine in PWH [21,22], but they lacked non-PWH as controls and had a limited follow-up period not exceeding one month. One study showed that GMTs of neutralizing antibodies against ancestral SARS-CoV-2 increased 4.8-fold from day 0 to day 15 after bivalent BA.4/5 booster vaccination [21]. The other study reported that a fourth dose enhanced ancestral and omicron-BA.5-specific neutralization modestly over three-dose levels [22].

In line with the antibody responses measured in other populations [18], antibody levels observed in our study were substantially lower in participants with vector-based versus mRNA-based primary vaccination. Although the functionality of antibodies might differ between participants with different types of primary vaccination regimens, and neutralizing antibodies were not measured in this study, we assumed that the similar GMRs of S1-specific antibodies in PWH with vector-based versus mRNA-based primary vaccination indicated that they probably derived equal immunological benefit from bivalent BA.1 booster vaccination. Similar to the general population [23], PWH who reported prior COVID-19 had higher antibody levels at baseline, but not after bivalent BA.1 booster vaccination.

The kinetics of the SARS-CoV-2-specific T-cell responses were characterized by a peak on day 7, followed by a rapid decline until day 28 and a more gradual decline until day 180. T-cell responses seemed to diverge between PWH and non-PWH over time, with lower T-cell responses observed in PWH on day 180, coinciding with more frequent intercurrent COVID-19 in non-PWH as possible explanation. In PWH, the median IFN-γ levels on day 180 were just under the cut-off for test positivity. Another study in PWH found a numerical but non-significant increase in the T-cell response from baseline to 15 days after bivalent vaccination [21]. These observations support a more rapid waning of the cellular response compared to the antibody response following bivalent BA.1 vaccination.

Regarding SARS-CoV-2-specific cytokine responses, we found an increase in both the Th_1_-type cytokines IFN-γ and IL-2 and in the Th_2_-type cytokine IL-4 from day 0 to day 28 after bivalent BA.1 vaccination. Clustering of the Th_1_-type and Th_2_-type cytokines distinguished three clusters within PWH, without apparent Th_1_/Th_2_ cytokine imbalances. An imbalanced cytokine response has been associated with lower antibody levels and neutralizing antibodies after a second COVID-19 vaccination in patients with renal diseases [24]. In contrast with the study in patients with renal diseases, we found no association between our cluster subgroups and antibody levels. While a substantial proportion of the patients with renal diseases did not show seroconversion at that time, all our participants had positive antibody levels. These results suggest that a Th_1_/Th_2_ cytokine balance, more than absolute cytokine concentrations, relates to antibody development against SARS-CoV-2. However, it should be noted that the three cluster subgroups were small, with only eight participants in cluster 1, and that these conclusions might not be applicable to more immunocompromised PWH.

Our study had some other limitations. Our participants mostly had a normal CD4^+^ T-cell count (>500 cells/μl), which prevents the extrapolation of the results to PWH with a CD4^+^ T-cell count <250 cells/μl. Indeed, nine participants had a CD4^+^ T-cell count <500 cells/μl, of whom one < 250 cells/μl, but none of them showed clinical signs of cellular immunodeficiency. Lower vaccine responses in PWH with poor immune recovery can therefore not be excluded. Moreover, we did not perform functional antibody tests. Antibody levels have been shown to correlate well with neutralization. This correlation has been described for the ancestral SARS-CoV-2 [25,26], but was also demonstrated for more recent variants like BA.1, BA.5 and XBB.1.5, even after bivalent BA.1 vaccination [26]. Furthermore, cytokine concentrations were not measured in non-PWH. We anticipated that the probability of observing differences in cytokine profiles between PWH and non-PWH was low, because we did not observe a Th_1_/Th_2_ cytokine imbalance, as was seen in kidney transplant patients after vaccination [24]. Additionally, PWH and non-PWH with a vector-based prime received different primary vaccine types, ChAdOx1-S vs. Ad26.COV2.S. Although research comparing immunogenicity after booster vaccination in participants with a ChAdOx1-S-based versus Ad26.COV2.S-based primary vaccination is lacking, the largely comparable antibody responses suggested no major influence of the vector priming platform used. Sex distribution was also unbalanced, but so far, differences in immunogenicity after COVID-19 vaccination between men and women have not been reported. Finally, our study did not assess immune responses beyond a half-year horizon. However, the consistently similar humoral responses between PWH and non-PWH provide confidence that responses will not diverge during longer follow-up.

In conclusion, antibody responses were comparable between PWH and non-PWH up to 180 days after a bivalent BA.1 booster vaccine. This suggests that well treated PWH receive a comparable immunological benefit from the COVID-19 booster vaccination schedule as the general public, although the faster waning of SARS-CoV-2-specific T-cell responses needs correlation to clinical outcomes in order to determine whether COVID-19 booster vaccines should be prioritized in PWH.

## Acknowledgements

We would like to thank all study participants.

Authors’ contributions:

Conceptualization: M.J.J., N.H.T., D.G., R.D.dV., C.H.G.vK., S.B., B.J.A.R., K.B., P.H.M.vd.K., A.H.E.R., C.R.

Formal analysis: M.J.J., P.M.A.

Funding acquisition: R.D.dV., B.J.A.R., K.B., P.H.M.vd.K., A.H.E.R, C.R.

Investigation: all authors.

Methodology: M.J.J., N.H.T., D.G., R.D.dV., C.H.G.vK., K.S.H., R.S.G.S., S.B., P.M.A., B.E.H., B.J.A., K.B., P.H.M.vdK., A.H.E.R., C.R.

Project administration: M.J.J., N.H.T.

Supervision: R.D.dV., C.H.G.vK., B.E.H., B.J.A.R., K.B., P.H.M.vdK., A.H.E.R., C.R.

Validation: M.J.J., N.H.T., D.G., R.D.dV., C.H.G.vK., B.J.A., K.B., P.H.M.vdK., A.H.E.R., C.R.

Visualization: M.J.J., P.M.A.

Writing – original draft: M.J.J., B.J.A.R., K.B., R.D.dV., C.H.G.vK., P.H.M.vdK., A.H.E.R., C.R.

Writing – review & editing: all authors contributed to reviewing and editing of the manuscript.

Previous presentations of this work: This work was presented at the 19th European AIDS Conference (EACS 2023).

### Conflicts of interest

Conflicts of interest and source of funding: This COVIH-BOOST-2 study was supported by the Dutch Organization for Health Research and Development (ZonMw) [10430072010008]. The SWITCH-ON study was supported by ZonMw [10430072110001]. D.G. and R.D.dV. were supported by Health∼Holland [EMCLHS20017] co-funded by the PPP Allowance made available by the Health∼Holland, Top Sector Life Sciences & Health, to stimulate public−private partnerships. R.D.dV. is listed as inventor of the fusion inhibitory lipopeptide [SARS_HRC_-PEG_4_]_2_-chol on a provisional patent application. K.S.H. has received support for attending meetings and travel from Gilead. B.E.H. has received research grants from Intercept, Cymabay, Mirum, Ipsen, Albireo, Calliditas and receives honoraria for advisory boards and consultancy from additional Chemomab and Pliant. B.J.A.R. declares the receipt of research grants from Gilead and MSD and honoraria for advisory boards from AstraZeneca, Roche, Gilead and F2G. K.B. has received research and educational grants from ViiV and Gilead, and consulting fees for advisory boards from ViiV, Gilead, MSD, and AstraZeneca. A.R. has received grants from the Bill and Melinda Gates Foundation and the Leids Universitair Fonds, participated on the board of an investor-initiated clinical trial on convalescent plasma for COVID-19, and is the chief editor of the Dutch Journal of Infectious Diseases and a member of the European Medicines Agency expert group on vaccines. C.R. has received research grants from ViiV, Gilead, ZonMW, AIDSfonds, Erasmus MC and Health∼Holland and honoraria for advisory boards from Gilead and ViiV. For the remaining authors none were declared.

## Supplementary Material

Supplemental Digital Content
