# Supplementary material for: Immunogenicity of a bivalent BA.1 COVID-19 booster vaccine in people with HIV in the Netherlands
Source: AIDS. 2024 May 28;38(9):1355–65. doi: 10.1097/QAD.0000000000003933 (PMC11216395; doi:10.1097/QAD.0000000000003933)
Supplement: Supplemental Digital Content [file aids-38-1355-s001.docx]

**Supplementary Materials**

**Immunogenicity of a bivalent BA.1** **COVID-19 booster vaccine in people with HIV in the Netherlands**

*Running head: Bivalent COVID-19 booster vaccination in PWH*

Marlou J. JONGKEES^1^ MD, Ngoc H. TAN^2^ MPharm, Daryl GEERS^3^ MSc, Rory D. DE VRIES^3^ PhD, Corine H. GEURTSVANKESSEL^3^ MD PhD, Kathryn S. HENSLEY^1^ MD, Roos S.G. SABLEROLLES^2^ MD, Susanne BOGERS^3^ MSc, Lennert GOMMERS^3^ BSc, Blerdi BLAKAJ^4^ BSc, Pedro MIRANDA AFONSO^5^ MSc, Bettina E. HANSEN^5,6,7^ PhD, Bart J.A. RIJNDERS^1^ MD PhD, Kees BRINKMAN^8^ MD PhD, P. Hugo M. VAN DER KUY^2^ PharmD PhD, Anna H.E. ROUKENS^9#^ MD PhD, Casper ROKX^1#^ MD PhD

*# These authors contributed equally*

*1. Department of Internal Medicine, Section Infectious Diseases, and Department of Medical Microbiology and Infectious Diseases, Erasmus University Medical Centre, Rotterdam, the Netherlands*

*2. Department of Hospital Pharmacy, Erasmus University Medical Centre, Rotterdam, the Netherlands*

*3. Department of Viroscience, Erasmus University Medical Centre, Rotterdam, the Netherlands*

*4. Department of Medical Oncology, Erasmus University Medical Centre, Rotterdam, the Netherlands*

*5. Department of Epidemiology and Biostatistics, Erasmus University Medical Centre, Rotterdam, the Netherlands*

*6. Institute of Health Policy, Management and Evaluation, University of Toronto, Toronto, Canada*

*7. Toronto Centre for Liver Disease, Toronto General Hospital University Health Network, Toronto, Canada*

*8. Department of Internal Medicine and Infectious Diseases, OLVG Hospital, Amsterdam, the Netherlands*

*9. Department of Infectious Diseases, Leiden University Medical Centre, Leiden, the Netherlands*

**Table of Contents**

[**Supplementary Tables** 4](#_Toc165998578)

[Supplementary Table 1. STROBE Statement 4](#_Toc165998579)

[Supplementary Table 2. Solicited adverse reactions in PWH 9](#_Toc165998580)

[**Supplementary Figures** 10](#_Toc165998581)

[Supplementary Figure 1. SARS-CoV-2 spike (S1)-specific antibody response after bivalent BA.1 booster vaccination in PWH with an mRNA-based vs vector-based prime 10](#_Toc165998582)

[Supplementary Figure 2. SARS-CoV-2-specific T-cell response after bivalent BA.1 booster vaccination in PWH with an mRNA-based vs vector-based prime 11](#_Toc165998583)

[Supplementary Figure 3. SARS-CoV-2-specific T-cell response after stimulation of whole blood with antigen 1 and 3 in PWH 13](#_Toc165998584)

[Supplementary Figure 4. Additional SARS-CoV-2-specific cytokine response in PWH after bivalent BA.1 vaccination 14](#_Toc165998585)

[Supplementary Figure 5. Three distinguished cluster profiles in PWH based on the Th_1_-type and Th_2_-type cytokine concentrations 15](#_Toc165998586)

[Supplementary Figure 6. SARS-CoV-2 spike (S1)-specific antibody response and SARS-CoV-2-specific T-cell response in PWH stratified based on the found cytokine cluster profiles 16](#_Toc165998588)

# Supplementary Tables

## Supplementary Table 1. STROBE Statement

|  | **Item No** | **Recommendation** | **Section** |
| --- | --- | --- | --- |
| **Title and abstract** | 1 | (*a*) Indicate the study’s design with a commonly used term in the title or the abstract | Abstract |
|  |  | (*b*) Provide in the abstract an informative and balanced summary of what was done and what was found | Abstract |
| **Introduction** | | |  |
| Background/rationale | 2 | Explain the scientific background and rationale for the investigation being reported | Introduction, paragraph 1, 2, and 3 |
| Objectives | 3 | State specific objectives, including any prespecified hypotheses | Introduction, paragraph 4 |
| **Methods** | | |  |
| Study design | 4 | Present key elements of study design early in the paper | Study design and participants |
| Setting | 5 | Describe the setting, locations, and relevant dates, including periods of recruitment, exposure, follow-up, and data collection | Study design and participants; Clinical procedures |
| Participants | 6 | (*a*) Give the eligibility criteria, and the sources and methods of selection of participants. Describe methods of follow-up | Study design and participants; Clinical procedures |
|  |  | (*b*) For matched studies, give matching criteria and number of exposed and unexposed | Study design and participants |
| Variables | 7 | Clearly define all outcomes, exposures, predictors, potential confounders, and effect modifiers. Give diagnostic criteria, if applicable | Outcomes |
| Data sources/ measurement | 8* | For each variable of interest, give sources of data and details of methods of assessment (measurement). Describe comparability of assessment methods if there is more than one group | Clinical procedures; Laboratory procedures |
| Bias | 9 | Describe any efforts to address potential sources of bias | Study design and participants |
| Study size | 10 | Explain how the study size was arrived at | Sample size and statistical analysis plan, paragraph 1 |
| Quantitative variables | 11 | Explain how quantitative variables were handled in the analyses. If applicable, describe which groupings were chosen and why | Outcomes; Sample size and statistical analysis plan, paragraph 2 |
| Statistical methods | 12 | (*a*) Describe all statistical methods, including those used to control for confounding | Sample size and statistical analysis plan |
|  |  | (*b*) Describe any methods used to examine subgroups and interactions | Sample size and statistical analysis plan, paragraph 2 |
|  |  | (*c*) Explain how missing data were addressed | Sample size and statistical analysis plan, paragraph 2 |
|  |  | (*d*) If applicable, explain how loss to follow-up was addressed | Not applicable |
|  |  | (*e*) Describe any sensitivity analyses | Not applicable |
| **Results** | | |  |
| Participants | 13* | (a) Report numbers of individuals at each stage of study—eg numbers potentially eligible, examined for eligibility, confirmed eligible, included in the study, completing follow-up, and analysed | Baseline characteristics |
|  |  | (b) Give reasons for non-participation at each stage | Baseline characteristics |
|  |  | (c) Consider use of a flow diagram | Not applicable |
| Descriptive data | 14* | (a) Give characteristics of study participants (eg demographic, clinical, social) and information on exposures and potential confounders | Baseline characteristics, Table 1 |
|  |  | (b) Indicate number of participants with missing data for each variable of interest | Footnote of figures |
|  |  | (c) Summarise follow-up time (eg, average and total amount) | Baseline characteristics |
| Outcome data | 15* | Report numbers of outcome events or summary measures over time | Humoral response in PWH versus non-PWH; T-cell response in PWH versus non-PWH |
| Main results | 16 | (*a*) Give unadjusted estimates and, if applicable, confounder-adjusted estimates and their precision (eg, 95% confidence interval). Make clear which confounders were adjusted for and why they were included | Humoral response in PWH versus non-PWH; T-cell response in PWH versus non-PWH |
|  |  | (*b*) Report category boundaries when continuous variables were categorized | Not applicable |
|  |  | (*c*) If relevant, consider translating estimates of relative risk into absolute risk for a meaningful time period | Not applicable |
| Other analyses | 17 | Report other analyses done—eg analyses of subgroups and interactions, and sensitivity analyses | Hybrid immunity in PWH; Cytokine response in PWH; Solicited reactions |
| **Discussion** | | |  |
| Key results | 18 | Summarise key results with reference to study objectives | Discussion paragraph 1 |
| Limitations | 19 | Discuss limitations of the study, taking into account sources of potential bias or imprecision. Discuss both direction and magnitude of any potential bias | Discussion, paragraph 6 |
| Interpretation | 20 | Give a cautious overall interpretation of results considering objectives, limitations, multiplicity of analyses, results from similar studies, and other relevant evidence | Discussion, paragraph 2 – 5 |
| Generalisability | 21 | Discuss the generalisability (external validity) of the study results | Discussion, paragraph 5 and 6 |
| **Other information** | | |  |
| Funding | 22 | Give the source of funding and the role of the funders for the present study and, if applicable, for the original study on which the present article is based | Conflicts of Interest and Source of Funding |

*Give information separately for exposed and unexposed groups

**Note**: An Explanation and Elaboration article discusses each checklist item and gives methodological background and published examples of transparent reporting. The STROBE checklist is best used in conjunction with this article (freely available on the Web sites of PLoS Medicine at http://www.plosmedicine.org/, Annals of Internal Medicine at http://www.annals.org/, and Epidemiology at http://www.epidem.com/). Information on the STROBE Initiative is available at http://www.strobe-statement.org.

| Supplementary Table 2. Solicited adverse reactions in PWH | | | | |
| --- | --- | --- | --- | --- |
|  | *None* | *Mild* | *Moderate* | *Severe* |
| **Local side-effects** | | | | |
| Rash at vaccination site | 19 (61%) | 10 (32%) | 2 (7%) | 0 |
| Pain at vaccination site | 13 (32%) | 17 (55%) | 1 (3%) | 0 |
| **Systemic side-effects** | | | | |
| Muscle ache | 25 (81%) | 5 (16%) | 1 (3%) | 0 |
| Rash | 29 (97%) | 1 (3%) | 0 | 0 |
| Headache | 22 (71%) | 5 (16%) | 3 (10%) | 1 (3%) |
| Fever | 28 (90%) | 2 (6%) | 0 | 0 |

Solicited adverse reactions in PWH occurring within seven days after the administration of the bivalent BA.1 booster vaccine. The severity of solicited adverse reactions was measured as no symptoms at all, mild (symptoms present but no medication needed or functional impairment), moderate (medication needed, no functional impairment), or severe (impaired daily functioning). Values are count (%). Abbreviations: PWH, people with human immunodeficiency virus.

# Supplementary Figures


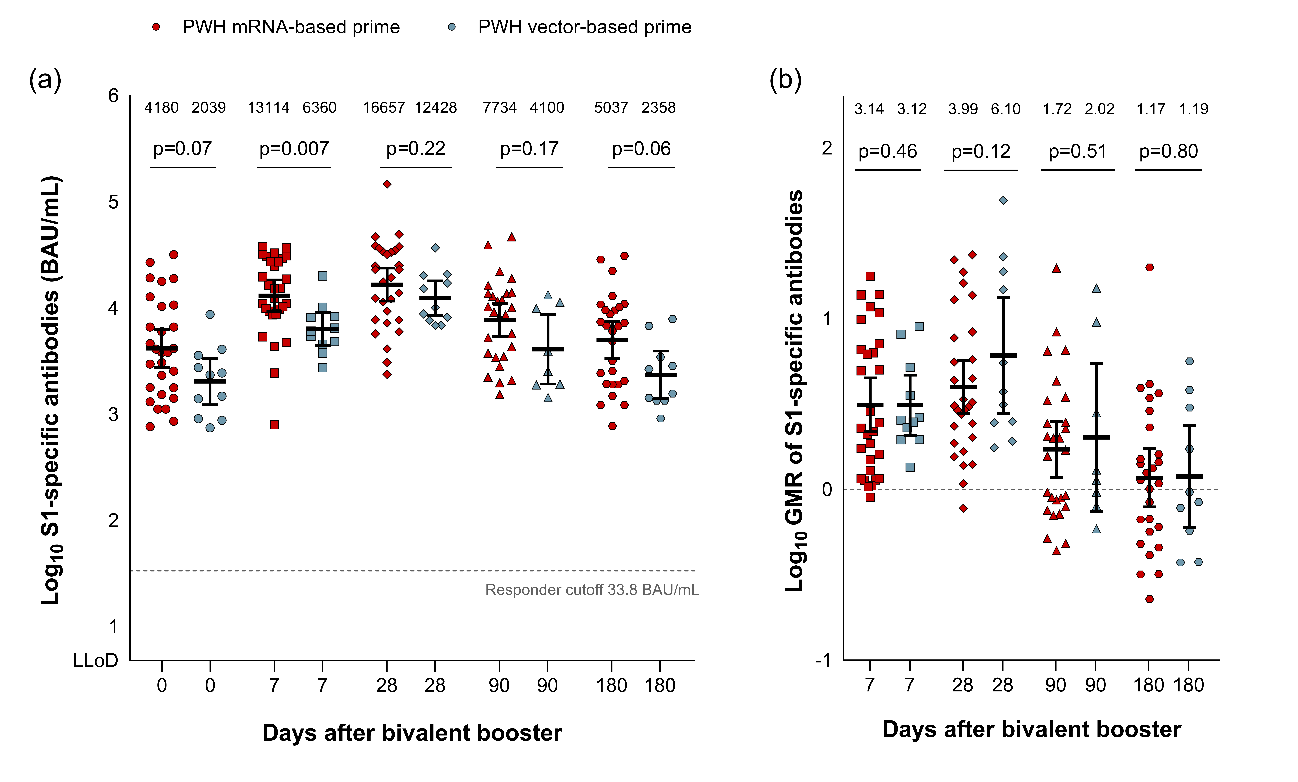


## Supplementary Figure 1. SARS-CoV-2 spike (S1)-specific antibody response after bivalent BA.1 booster vaccination in PWH with an mRNA-based vs vector-based prime

(a) Detection of ancestral S1-specific antibodies at baseline before bivalent BA.1 booster vaccination (day 0, indicated by circles), and on days 7 (squares), 28 (diamonds), 90 (triangles), and 180 (hexagons) after bivalent BA.1 booster vaccination in PWH with an mRNA-based prime (indicated in red) and PWH with a vector-based prime (indicated in blue). The numbers above the plots and the middle whiskers in the plot indicate the geometric mean titres per time point, while the lower and upper whiskers in the plot indicate the corresponding 95% confidence intervals. The lower limit of detection was set at 4.81 binding antibody units per millilitre (BAU/mL), and the cut-off responder value was set at 33.8 BAU/mL (horizontal dashed line). Comparisons between groups were performed using the independent *t*-test. (b) GMRs of S1-specific antibodies compared to day 0 on days 7, 28, 90, and 180 in PWH with an mRNA-based prime (indicated in red) and PWH with a vector-based prime (indicated in blue). The horizontal dashed line indicates a Log_10_ GMR of 0, corresponding to no difference in S1-specific antibodies. Comparisons between groups were performed using the independent *t*-test. Abbreviations: BAU, binding antibody units; GMR, geometric mean ratio; LLoD, lower limit of detection; non-PWH, people without human immunodeficiency virus; PWH, people with human immunodeficiency virus; S, spike.


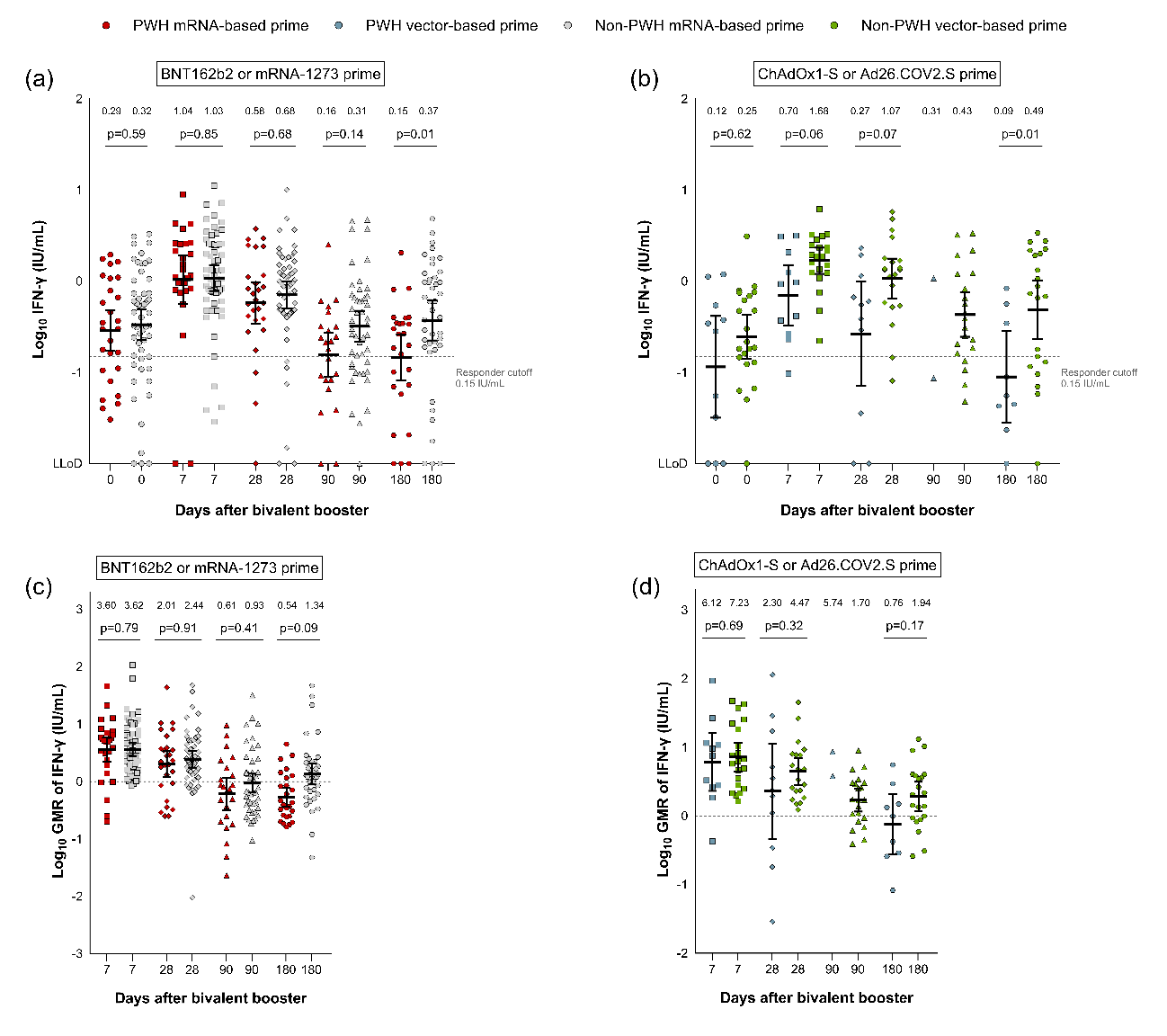


Supplementary Figure 2. SARS-CoV-2-specific T-cell response after bivalent BA.1 booster vaccination in PWH with an mRNA-based vs vector-based prime

Detection of IFN-γ (IU/mL) after the stimulation of whole blood with antigen 2 in coated QuantiFERON tubes at baseline before bivalent BA.1 booster vaccination (0 days, circles) and on days 7 (squares), 28 (diamonds), 90 (triangles), and 180 (hexagons) after bivalent BA.1 booster vaccination in participants with an mRNA-based prime (N=29 PWH and N=58 non-PWH; panel a) or a vector-based prime (N=11 PWH and N=22 non-PWH; panel b). The lower limit of detection was set at 0.01 IU/mL as per the manufacturer's instructions, and the cut-off responder value was set at 0.15 IU/mL (horizontal dashed line). The numbers above the plots and the middle whiskers in the plot indicate the geometric means per time point, while the lower and upper whiskers in the plot indicate the corresponding 95% confidence intervals. Comparisons between groups were performed using the independent *t*-test. A comparison between groups was not applied to the vector-based prime groups on day 90 due to the limited (N=2) IFN-γ results for PWH. GMRs of IFN-γ compared to day 0 on days 7, 28, 90, and 180 in PWH with an mRNA-based prime (panel c) or a vector-based prime (panel d). The horizontal dashed line indicates a Log_10_ GMR of 0, corresponding to no difference in IFN-γ. Comparisons between groups were performed using the independent *t*-test. A comparison between groups was not applied to the vector-based prime groups on day 90 due to the limited (N=2) IFN-γ results for PWH. Abbreviations: GMR, geometric mean ratio; IU, international units; LLoD, lower limit of detection; PWH, people with human immunodeficiency virus.

**
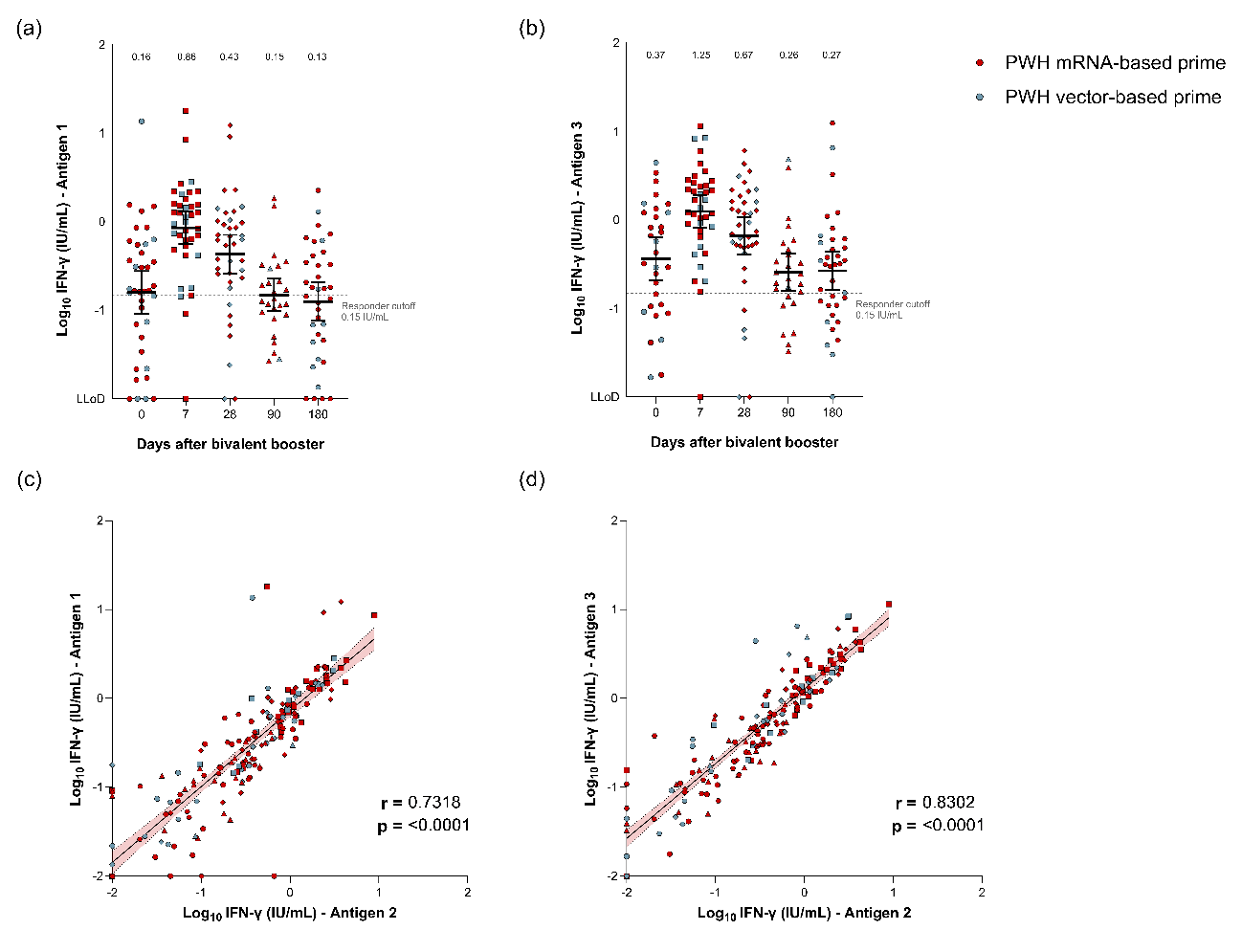
**

Supplementary Figure 3. SARS-CoV-2-specific T-cell response after stimulation of whole blood with antigen 1 and 3 in PWH

Detection of IFN-γ (IU/mL) after the stimulation of whole blood with antigen 1 (panel a) and antigen 3 (panel b) in coated QuantiFERON tubes at baseline before bivalent BA.1 booster vaccination (0 days, circles) and on days 7 (squares), 28 (diamonds), 90 (triangles), and 180 (hexagons) after bivalent BA.1 booster vaccination in PWH (N=40). The lower limit of detection was set at 0.01 IU/mL as per the manufacturer's instructions, and the cut-off responder value was set at 0.15 IU/mL (horizontal dashed line). The numbers above the plots and the middle whiskers in the plot indicate the geometric mean titres per time point, while the lower and upper whiskers in the plot indicate the corresponding 95% confidence intervals. Correlation of IFN-γ levels after stimulation of whole blood with antigens 2 and 1 (panel c) or antigens 2 and 3 (panel d) on all five time points. Correlations were evaluated by linear regression analysis on transformed data. Abbreviations: GMR, geometric mean ratio; IU, international units; LLoD, lower limit of detection; PWH, people with human immunodeficiency virus.


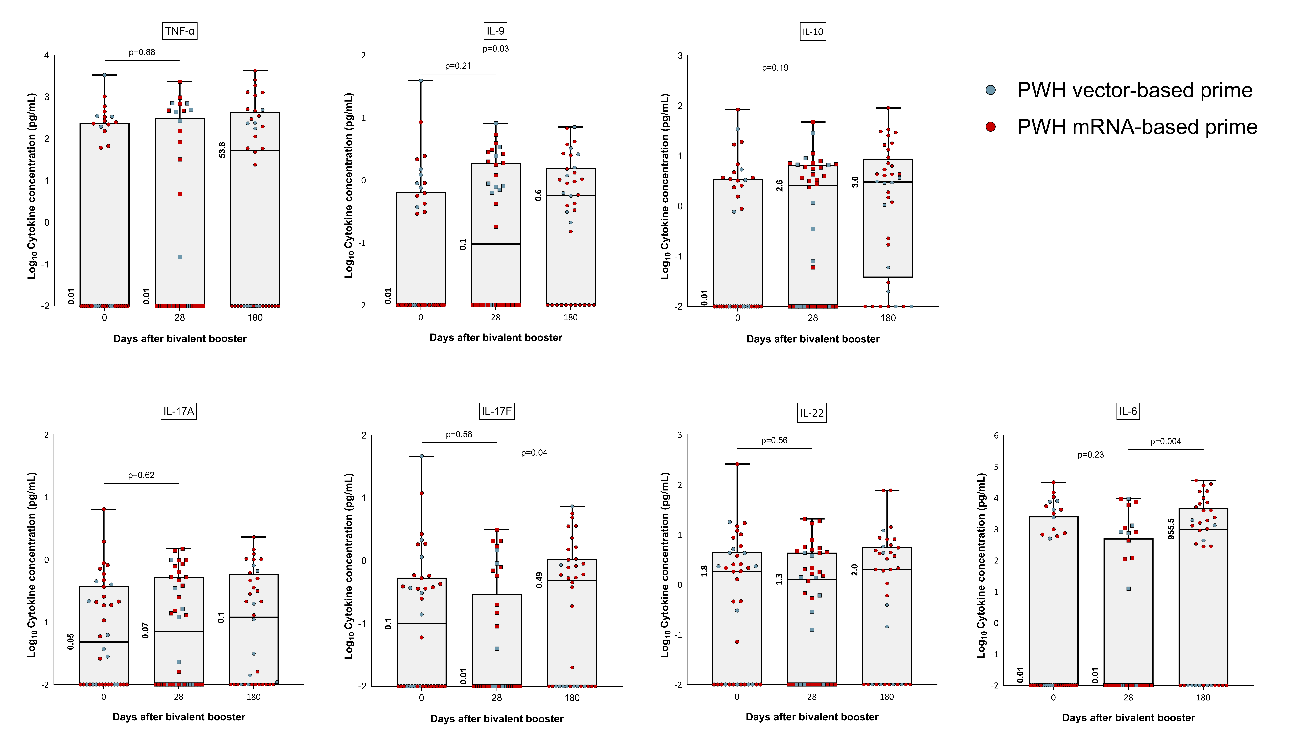


## Supplementary Figure 4. Additional SARS-CoV-2-specific cytokine response in PWH after bivalent BA.1 vaccination

Cytokine concentrations (pg/mL) for the seven additional cytokines (TNF-α, IL-9, IL-10, IL-17A, IL-17F, IL-22, IL-6) measured after stimulation of whole blood with antigen 2 in coated QuantiFERON tubes at baseline before bivalent BA.1 booster vaccination, and on day 28 and 180 after bivalent BA.1 vaccination in all PWH (N=40). The horizontal lines within the whiskers and numbers indicate the medians, and the tops and bottoms indicate the interquartile ranges. Wilcoxon matched-pairs signed rank tests were applied to the comparisons. Abbreviations: pg, picogram; PWH, people with human immunodeficiency virus.

## **Supplementary Figure 5. Three distinguished cluster profiles in PWH based on the Th_1_-type and Th_2_-type cytokine concentrations**

Heatmap of an unsupervised cluster analysis, using the Ward’s method, of centred and scaled IFN-γ, IL-2, IL-4, IL-5, and IL-13 cytokine concentrations in PWH on day 28 after bivalent BA.1 booster vaccination. Each column represents a single participant.

**
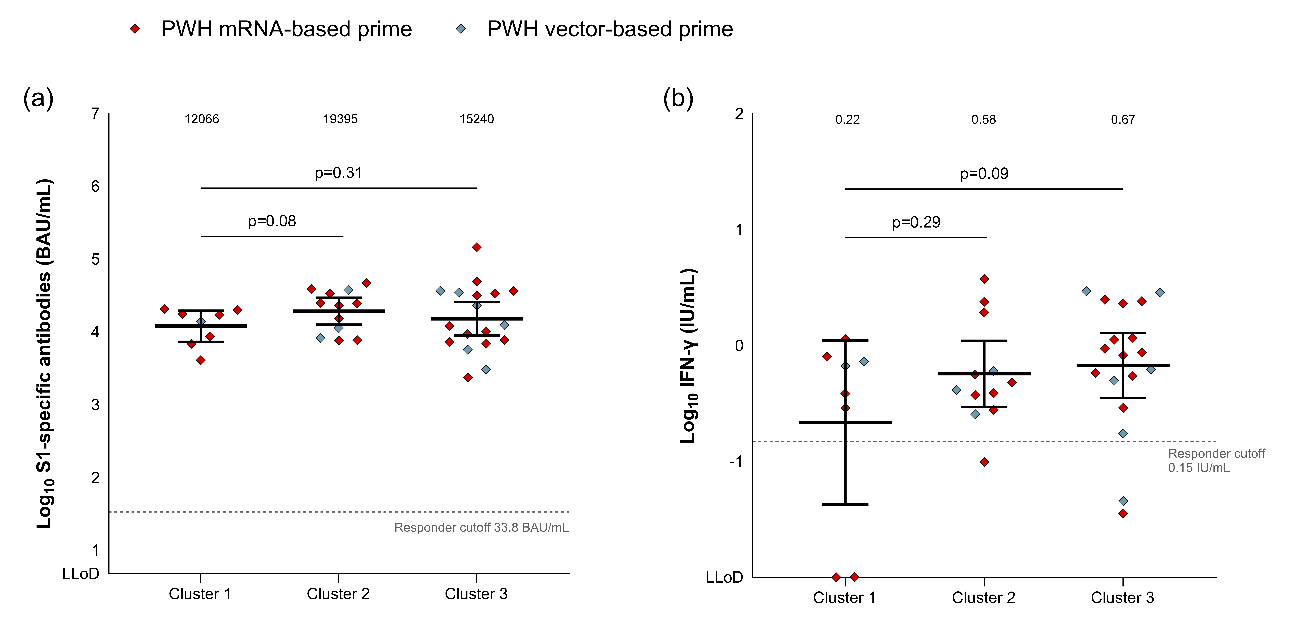
**

Supplementary Figure 6. SARS-CoV-2 spike (S1)-specific antibody response and SARS-CoV-2-specific T-cell response in PWH stratified based on the found cytokine cluster profiles

(a) Detection of ancestral S1-specific antibodies on day 28 in PWH with cytokine cluster profile 1, 2, or 3. The numbers above the plots and the middle whiskers in the plot indicate the geometric mean titres per time point, while the lower and upper whiskers in the plot indicate the corresponding 95% confidence intervals. The lower limit of detection was set at 4.81 binding antibody units per millilitre (BAU/mL), and the cut-off responder value was set at 33.8 BAU/mL (horizontal dashed line). Comparisons between groups were performed using the independent *t*-test. (b) Detection of IFN-γ (IU/mL) after the stimulation of whole blood with antigen 2 in coated QuantiFERON tubes on day 28 in PWH with cytokine cluster profile 1, 2, or 3. The numbers above the plots and the middle whiskers in the plot indicate the geometric means per time point, while the lower and upper whiskers in the plot indicate the corresponding 95% confidence intervals. The lower limit of detection was set at 0.01 IU/mL as per the manufacturer's instructions, and the cut-off responder value was set at 0.15 IU/mL (horizontal dashed line). Comparisons between groups were performed using the independent *t*-test. Abbreviations: BAU, binding antibody units; IU, international units; LLoD, lower limit of detection; PWH, people with human immunodeficiency virus; S, spike.
